# Equivalent method for calculating internal blast loads in cylindrical lattice shell structure

**DOI:** 10.1038/s41598-025-13066-4

**Published:** 2025-07-29

**Authors:** Fu Shiqi, Gao Xuanneng

**Affiliations:** 1 College of Engineering, Fujian Jiangxia University, Fuzhou, 350100 Fujian PR China; 2 College of Civil Engineering, Huaqiao University, Xiamen, 361021 Fujian PR China; 3 Institute of Infrastructural Protection in Fujian Jiangxia University, Fuzhou, 350100 Fujian PR China

**Keywords:** Cylindrical lattice shell structure, Internal explosion, Shock waves, Equivalent method, Civil engineering, Scientific data

## Abstract

It is crucial to improve the calculation efficiency of internal blast loads of a long-span spatial steel structure. This study develops an equivalent model for such loads using a one-way inclined single-layer cylindrical lattice shell structure as a case study. First, ANSYS/LS-DYNA was used to simulate free-air blasts and benchmark against experimental data, with peak overpressure errors below 8%, confirming the modeling approach and material parameters. Next, a numerical model of the cylindrical lattice shell structure under internal explosion was generated via the same modelling method and material parameters. The simulation results indicated that the internal explosion overpressure differed from that of free-air blasts, exhibited pronounced reflection and convergence effects, and was no longer related to the scaled distance. On this basis, an equivalent model combining a standard overpressure distribution with correction factors for reflection and convergence was formulated. Validation against two additional case studies demonstrated that the model provides conservative predictions, with average errors of 9.38% and 7.47% relative to detailed simulations. The proposed equivalent model therefore offers a rapid, reliable tool for the preliminary assessment of internal blast loads in similar spatial structures.

## Introduction

Long-span spatial steel structures are ubiquitous in modern cities and often serve as landmark buildings. An explosion in such a structure can cause significant casualties and economic damage. Consequently, research into their blast resistance is both theoretically and practically important and has attracted widespread attention from scholars worldwide.

The blast load serves as the basis for blast resistance research. Many researchers have experimentally investigated the overpressure of blasts to derive empirical formulas and even contribute to the formation of specifications. Baker^1^ established a systematic theory of air blasts on the basis of experiments. Henrych et al. and Moszynski et al.^2,3^ carried out numerous air blast tests and derived empirical formulas for calculating blast loads. On the basis of these studies, the relevant codes were published in Western countries. In these codes, the blast load can be classified into free-field blasts and confined blasts, and methods for calculating the overpressure of shock waves are provided^4–6^. However, codes and empirical formulas have specific application scopes. Under complex working conditions, specific analyses must be carried out. For example, to study the blast load in tunnels and caverns, Smith^7^ conducted internal explosion tests in cubic caverns and tunnels with partial openings and measured the overpressure of shock waves at different locations within the structure. Scheklinski^8^ designed and carried out internal explosion experiments on scaled models to study blast loading in connected rooms and obtained empirical formulas for overpressure, positive pressure duration, specific impulses, and quasistatic gas pressure.

Compared with expensive blast tests, numerical simulations are more cost effective in blast resistance research. Chapman^9^ utilized AUTODYN to study the propagation laws of shock waves and the blast load on structures and then compared the simulation results with the experimental results to demonstrate the accuracy of the numerical simulations. Langlet^10^ employed ANSYS/LS-DYNA to simulate the reflection of shock waves from rigid cylindrical shells, and empirical formulas were derived on the basis of the simulation data. Li^11,12^ used ANSYS/LS-DYNA to establish a numerical model of a tunnel under internal explosion and carried out simulation calculations. After verifying the correctness of the simulation results, the influences of factors such as the mesh size and tunnel length on the shock waves were analysed. Wu and Xu^13,14^ conducted numerical simulations to investigate the effects of explosion vents on internal blast load characteristics. Hao^15–18^ developed numerical models to simulate the blast response characteristics of critical building components, including walls, columns, and glazing systems. Through systematic validation against data, both the blast load distribution and structural response mechanisms were investigated. The abovementioned studies clearly demonstrated that numerical simulations can reproduce many details of blast shock waves. However, the modelling and computation processes are time-consuming, which makes them inefficient and less suitable for large-scale applications.

To increase the efficiency of calculating blast loads in engineering, researchers have developed equivalent calculation models for blast loads on the basis of test or simulation results. For example, Ding^19^ simulated the overpressure of shock waves in plate lattice shell structures under internal explosion, studied the distribution laws of overpressure, and proposed a simplified calculation method for internal blast loads. By simulating the propagation laws of shock waves in tunnels under internal explosions, Yang^20,21^ derived the dynamic coefficient of internal explosion based on the principle of identical structural responses and established an equivalent calculation method for internal blast loads using the dynamic coefficient. Ma^22,23^ established a prediction model for internal blast loads in spherical lattice shell structures to rapidly calculate the overpressure of shock waves and specific impulses.

In this work, an efficient equivalent model capable of reflecting the characteristics of shock waves under internal explosion is established. First, ANSYS/LS-DYNA is used to establish a numerical model of an air blast. The rationality and reliability of the modelling method and material parameters could be verified by comparing the data from the air blast tests with the simulation results. Next, with the same modelling method and material parameters, a numerical model of the cylindrical lattice shell structure under internal explosion can be established. Then, the propagation laws and overpressure distributions of shock waves under internal explosion can be analysed, and different parameters can be discussed. Finally, an equivalent method for calculating internal blast loads, which consists of a standard distribution and correction factors, can be established. Its applicability can be verified by two examples.

## Numerical simulation and blast loads

ANSYS/LS-DYNA is an explicit dynamic analysis software. Its ALE (arbitrary Lagrange‒Euler) algorithm combines the advantages of the Lagrange and Euler algorithms, which makes it very suitable for dealing with the fluid‒structure interaction between the blast shock waves and the obstacle^24^. In this section, the ALE algorithm is used to calculate the shock waves of an air blast, the properties of the shock waves are discussed, and the accuracy of the modelling method and material parameter selection can be verified by comparing the data from the air blast tests with the simulation results.

### Simulation for air blasts

**Fig. 1:**
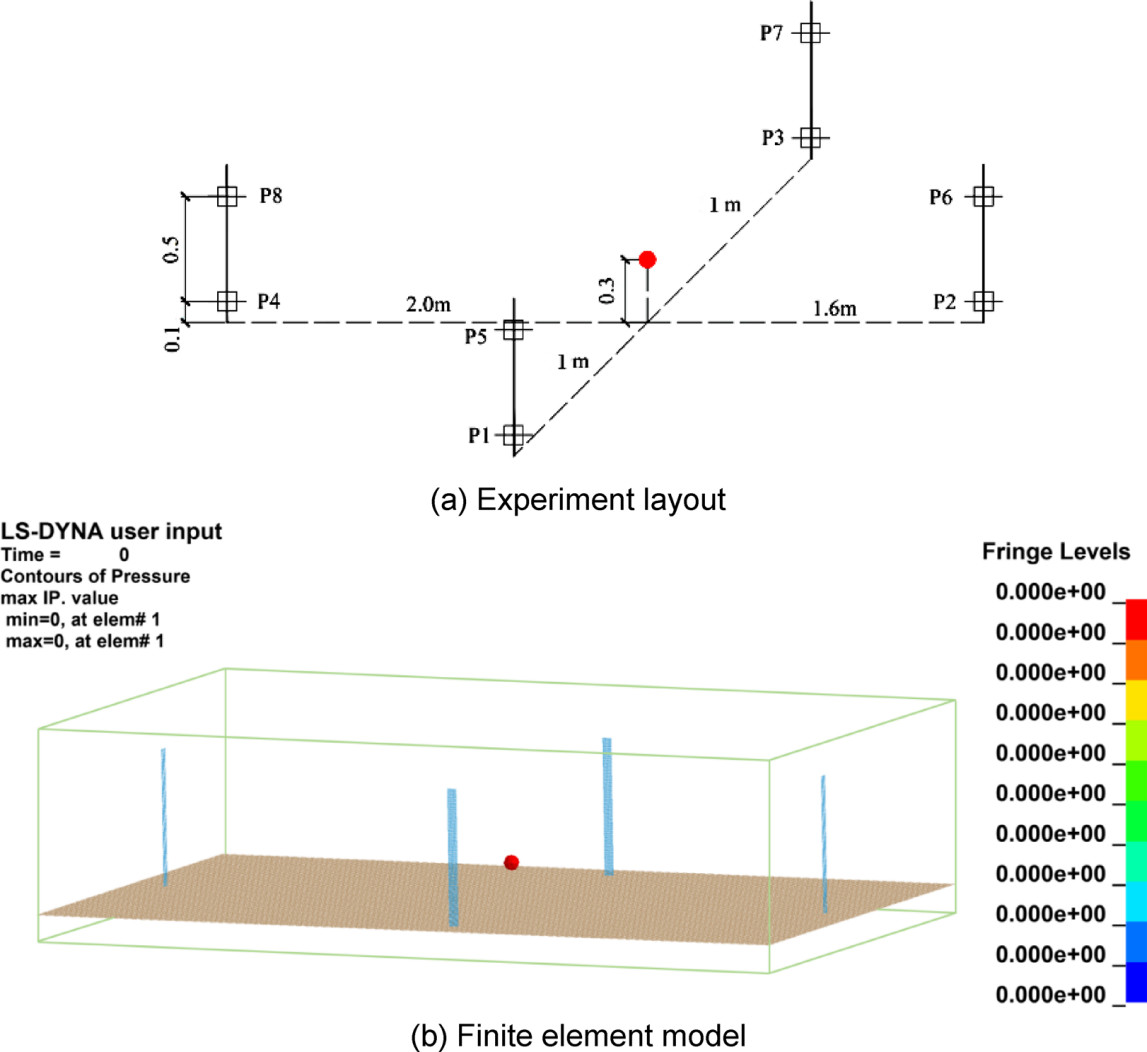
Experiment layout and numerical model of air blast.

In accordance with air blast tests from the literature^25,26^, a corresponding numerical model was established via ANSYS/LS-DYNA. As shown in Fig. 1, the numerical model of the air blast test consisted of a sensor mounting frame (angle iron), ground, explosive and air. Among them, the angle iron and the ground were stimulated by Shell163, and the ground was defined as rigid to consider the reflection effect of the angle iron and the ground on the shock waves^27–29^. The air was simulated by Solid164, the size was 4.3 × 2.6 × 1.1 m, and the grid size was divided into 0.025 × 0.025 × 0.025 m. The boundary was defined as the transmission boundary to simulate an explosion in an infinite area^27–29^. The total duration of the simulation is set to 0.01 s, discretized into 200 steps with a time increment of 5 × 10^− 5^ s per step.

Q235 steel was used for angle iron, and the modified Johnson‒Cook constitutive relation was selected, which considers the strain rate effect and strain strengthening effect of steel subjected to high-speed impact loading^30^. Specifically, it is shown in Eq. (1):

1 $$\sigma =({{\text{A}}_{\text{1}}}+{{\text{A}}_{\text{2}}}{\varepsilon ^{\text{n}}})(1+{{\text{A}}_{\text{3}}}\ln {\dot {\varepsilon }^*})(1 - {T^{*{{\text{n}}_{\text{t}}}}})$$

where $\sigma$, $\varepsilon$, ${\dot {\varepsilon }^{\text{*}}}$ and T* represent the equivalent flow stress, the equivalent plastic strain, the relative strain rate and the relative temperature, respectively. ${\dot {\varepsilon }^*}=\dot {\varepsilon }/{\dot {\varepsilon }_0}$, $\dot {\varepsilon }$ and ${\dot {\varepsilon }_0}$ are the strain rate and the strain rate under quasi-static loading, respectively. In addition, $1 - {T^{*{{\text{n}}_{\text{t}}}}}$represents the softening effect of temperature, which is not considered because the experiments or simulations are conducted at room temperature. In other words, T^*^ is zero and the value of n_t_ is arbitrary. That is, the following formula is obtained:

2 $$\sigma =({{\text{A}}_{\text{1}}}+{{\text{A}}_{\text{2}}}{\varepsilon ^{\text{n}}})(1+{{\text{A}}_{\text{3}}}\ln {\dot {\varepsilon }^*})$$

where, A_1_, A_2_, A_3_ and n are undetermined parameters that can be calibrated by experiment and are shown in Table 1^30^.

**Table 1:** Constitutive parameters of Q235^30^.

*ρ*_st_/kg·m^− 3^	E/GPa	*µ*	A_1_	A_2_	n	A_3_
7850	210	0.3	320.7556 × 10^6^	582.102 × 10^6^	0.3823	0.0255

*Note: *ρ*_st_, E and *µ* are density, Young’s modulus and Poisson’s ratio of Q235 steel, respectively.

The air was simulated by MAT_NULL, and the state equation *EOS_LINEAR_POLYNOMIAL was adopted, as follows.

3 $$\begin{gathered} {p_0}={C_0}+{C_1}\eta +{C_2}{\eta ^2}+{C_3}{\eta ^3}+({C_4}+{C_5}\eta +{C_6}{\eta ^2}) \cdot {E_{air}} \hfill \\ \eta =1/{V_{air}} - 1 \hfill \\ \end{gathered}$$

where, ${p_0}$ is the initial pressure of air, *C*_0_, *C*_1_, *C*_2_, *C*_3_, *C*_4_, *C*_5_ and *C*_6_ are the material parameters of air, and ${E_{air}}$ and ${V_{air}}$ the internal energy per unit volume and the relative volume of air, respectively^30^. The values are shown in Table 2.

**Table 2:** Material parameters of air^30^.

${\rho _{air}}/{\text{kg}} \cdot {{\text{m}}^{ - 3}}$	${C_0}$	${C_1}$	${C_2}$	${C_3}$	${C_4}$	${C_5}$	${C_6}$	${V_{0,air}}/{\text{J}} \cdot {{\text{m}}^{ - 3}}$	${E_{0,air}}$
1.29	0	0	0	0	0.4	0.4	0	2.5 × 10^5^	1.0

*Note: ${\rho _{air}}$, ${V_{0,air}}$ and ${E_{0,air}}$ are density, initial relative volume and initial internal energy of air, respectively.

The selected mass of TNT was 50 kg. The volume fraction method was used to define the explosion and the explosive location, which was located at a distance of 1.5 m from the ground in the centre of the air. The high-energy explosive model (*MAT_HIGH_EXPLOSIVE_BURN) was adopted, and the Jones‒Wilkins‒Lee state control equation is shown in Eq. (4).

4 $$P=A\left(1-\frac{\omega}{R_{1}V}\right)e^{-R_{1}V}+B\left(1-\frac{\omega}{R_{2}V}\right)e^{-R_{2}V}+\frac{\omega E_{0}}{V}$$

where, *p* is the pressure of the shock waves. *V* is the relative volume. ${E_0}$ is the initial internal energy of explosive. A, B, ${R_1}$, ${R_2}$ and $\omega$are input parameters, and are shown in Table 3.

**Table 3:** Material parameters of explosive^25–30^.

*ρ*/kg·m^− 3^	*D*/m·s^− 1^	*P*_CJ_/GPa	*A*/GPa	*B*/GPa	*R*_1_	*R*_2_	ω	*E*_0_/J·m^− 3^	*V*_0_
1630	6713	18.5	540.9	9.4	4.5	1.1	0.35	8.0 × 10^9^	1.0

*Note: *ρ*, *D* and *P*_*CJ*_ are explosive density, detonation velocity and detonation pressure, respectively.

### Characteristics of the shock wave

The experimental data were compared with the simulation results to verify the accuracy of the modelling method and material parameters. The experimental data were based on tests conducted with 80 g of emulsion explosive at points P2 and P6. The simulated overpressure corresponded to a calculation using 39.84 g of TNT. Notably, the mass conversion factor between TNT and the emulsion explosive was 0.498, which was derived by comparing scaled distances that produce the same overpressure.

**Fig. 2:**
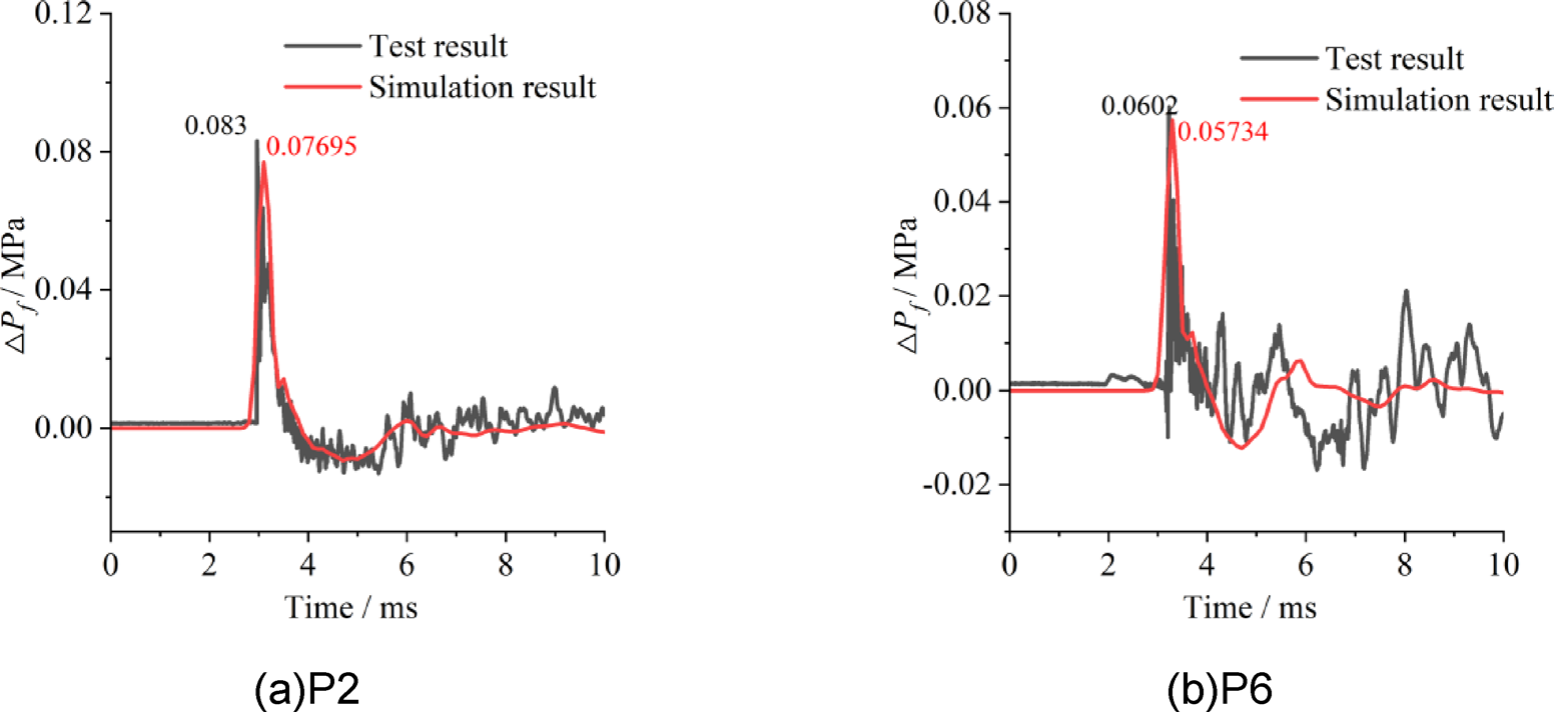
Comparison of overpressure between experiment and simulation.

The comparison results are shown in Fig. 2. The time–history curves of the shock waves from the air blast tests are consistent with those from the simulation, and the peak overpressures of the two waves agree very well. Compared with those obtained experimentally, the errors of the peak overpressure at P2 and P6 are 7.29% and 4.75%, respectively. The modelling method and material parameters described in Sect. "Simulation for air blasts" are reasonable, and the simulation results are reliable.

**Fig. 3:**
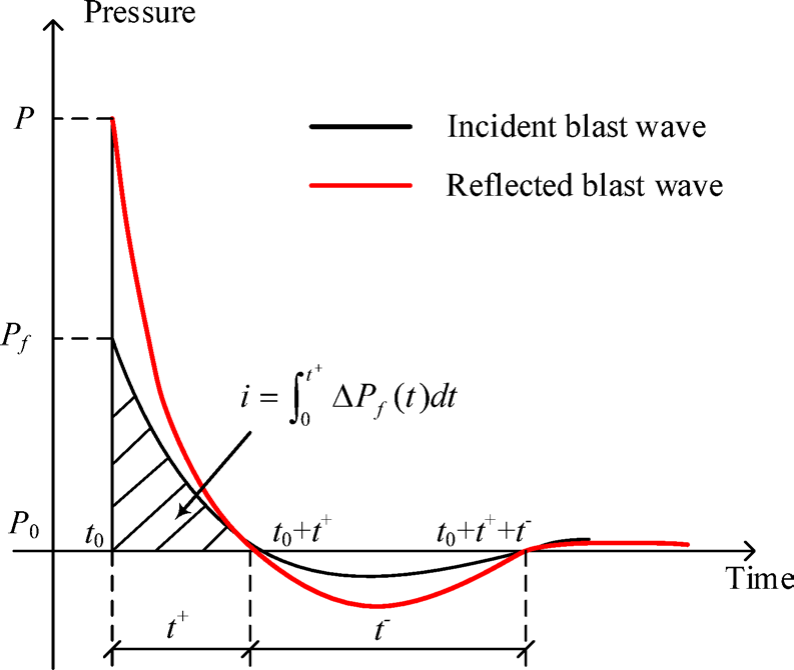
Time history curves of shock waves^31^.

On the basis of the experimental and simulation results, the time–history curve of shock waves can be theoretically described. As shown in Fig. 3, *P*_0_ is the standard atmospheric pressure. The shock wave generated by the explosion source arrives at the measurement point at time *t*_*0*_, causing an instantaneous pressure increase from *P*_0_ to *P*_*f*_. The difference between *P*_*f*_ and *P*_*0*_, Δ*P*_*f*_, is called the overpressure of shock waves. As the shock wave propagates forwards, the pressure continuously attenuates and returns to atmospheric pressure after time *t*^*+*^. Subsequently, the pressure continues to decrease to a negative value and returns to standard atmospheric pressure after time *t*^*−*^. The shock waves of a blast can be accurately described by determining the overpressure Δ*P*_*f*_, the positive pressure duration *t*^*+*^, and the specific impulse *i*.

### Typical blast loads

The damage effects generated by the shock waves of a blast can be evaluated by the overpressure criterion (P criterion), impulse criterion (I criterion), or overpressure-impulse criterion (P-I criterion). The so-called P criterion means that the damage effects can be evaluated by only overpressure^31^. Similarly, the I criterion and P-I criterion use specific impulses and overpressure-specific impulses, respectively, to evaluate the damage effects^31^. However, the I criterion neglects the influence of overpressure on structures, and the P-I criterion is more difficult to calculate. In most applications, the P criterion is used. Therefore, the overpressure of shock waves is used as the main parameter to determine the blast load.

Overpressure can be divided into incident overpressure and reflected overpressure^32^. In the past, empirical formulas for incident overpressure, such as those proposed by Henrych^2^, Sadovskyi^3^, and Bake^1^, were obtained via field experiments. However, there are varying degrees of errors among these empirical formulas. Wu performed a weighted average on these empirical formulas to reduce errors^33–35^. In addition, Wu compared the calculation results of numerical simulations and empirical formulas and reported that the numerical simulation results were consistent with the calculation results obtained via empirical formulas^33–35^. On the basis of numerical simulation results, Wu derived the following empirical formula^34^:

5 $$\Delta {P_f}=\frac{{0.0317}}{{\overline {R} }}+\frac{{0.3041}}{{{{\overline {R} }^2}}}+\frac{{0.1517}}{{{{\overline {R} }^3}}}\begin{array}{*{20}{c}} {}&{} \end{array}1 \leqslant \overline {R} \leqslant 6$$

where, $\bar {R}=R/\sqrt[3]{W}$ is scaled distance, whose unit is ${\text{m}} \cdot {\text{k}}{{\text{g}}^{ - 1/3}}$. *R* is the distance between the explosive centre and the target and *W* is the charge mass.

If the shock wave of a blast propagates to ground or buildings, a reflection phenomenon occurs, and the overpressure of shock waves acting on the ground or buildings is strengthened. The time–history curve of shock waves reflected from an infinite rigid plane is shown by the red line in Fig. 3^32^. The pressure of the reflected shock wave is approximately 28 times the incident pressure^32^. The blast load on buildings is mostly reflected overpressure, and the calculation results obtained via empirical formulas may underestimate the blast load.

## Internal blast loads in cylindrical lattice shell structure

### Numerical model of structure

**Fig. 4:**
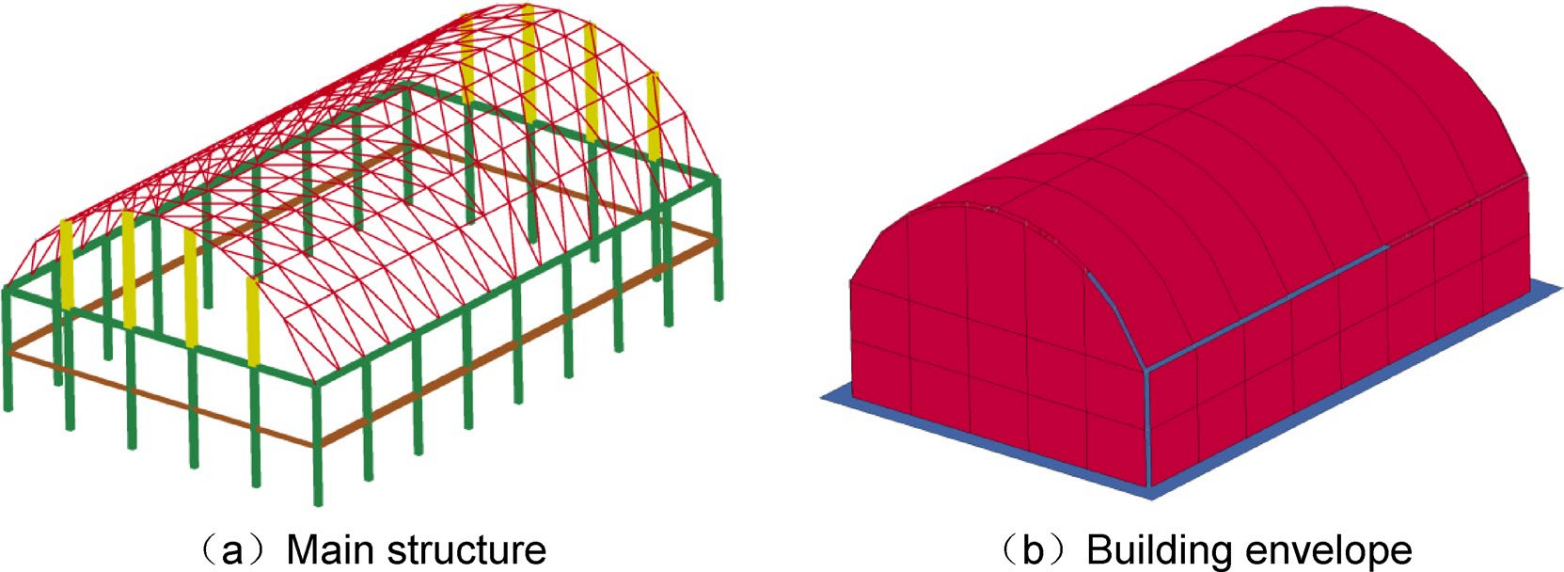
Numerical model of cylindrical lattice shell structure under internal explosion.

The same modelling method and material parameters as those used in Sect. "Simulation for air blasts" were used to establish a numerical model of the cylindrical lattice shell structure under internal explosion. As shown in Fig. 4, the numerical model included the main structure, building envelope, rigid ground, air domain and explosives.

The main structure was composed of an upper lattice shell structure and a lower support structure. The upper lattice shell structure was a single-layer cylindrical lattice shell, with a length of *l* = 40 m, a span of *b* = 30 m, and a rise of *f* = 10 m. The lower support structure was composed of square steel tubes with a height of *h* = 10 m, and the bottom end was fixed to the rigid ground. The building envelope was made of 2 mm steel plates, which were connected to the main structure by connection components with a length of 300 mm and a diameter of 35 mm. In modelling, all connection components are connected by common nodes. The main structure and connection components were simulated with Beam161 elements, and the building envelope was simulated with Shell163 elements.

A 180 kg sample of TNT was placed in the centre of the structure, 1.5 m above the ground. In the simulation, the ALE algorithm was used, and the interactions of the shock waves of the air blast with the building envelope and ground were considered.

### Over-pressure distribution in structure

**Fig. 5:**
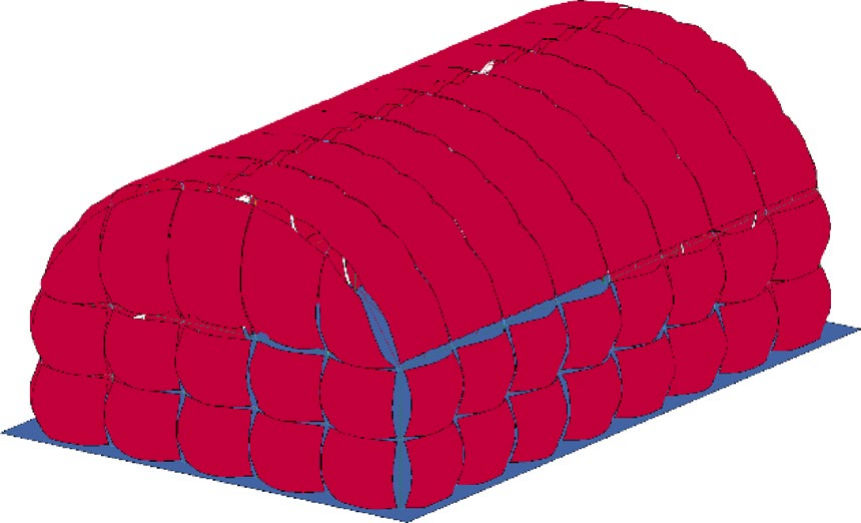
Damage to structure after explosion.

The total duration of the numerical calculation was 200 ms. The structural damage under internal explosion is shown in Fig. 5. The building envelope deformed to a certain extent but did not detach from the main structure. In other words, the shock waves do not escape, and the interaction between shock waves is completed within the structure.

**Fig. 6:**
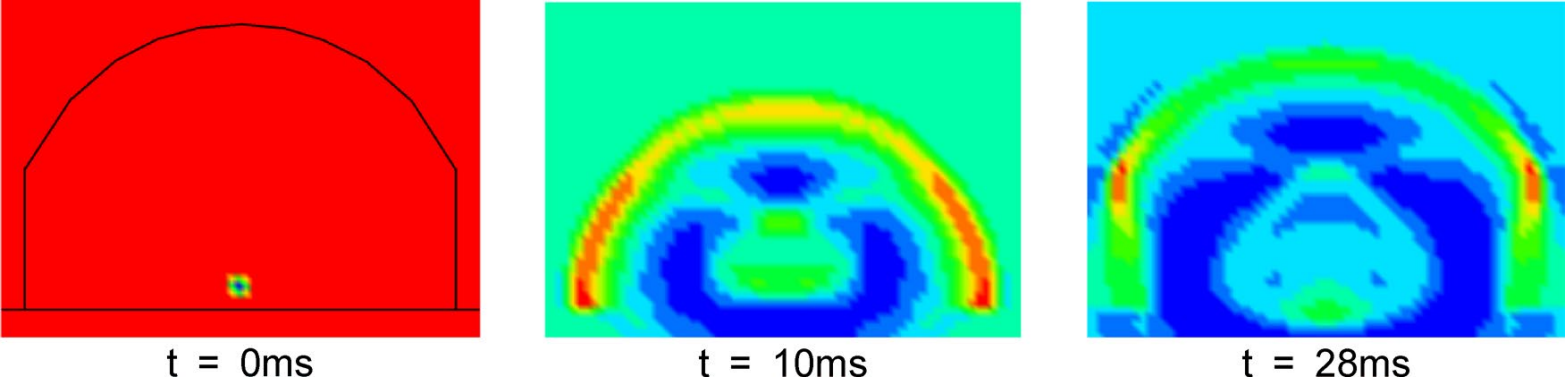
Propagation laws of shock waves under internal explosion.

To study the propagation laws of shock waves under internal explosion, the shock waves at 0 ms, 20 ms and 28 ms in a cross-section of the numerical model were extracted. As shown in Fig. 6, the shock waves first reach the building envelope and are reflected by the influence of the building envelope. The incident shock waves and the reflected shock waves interact via both additive and reducing effects. At 28 ms, the reflected shock waves converge at the corners of the structure, and the shock waves are amplified because of the convergence effects. The reflection phenomena and convergence effects for the shock waves of internal explosion clearly occur.

The overpressures of shock waves at the dome and corners of the structure were extracted and compared with the overpressures calculated via Eq. (5). The comparison results are displayed in Table 4. Owing to the influence of reflection phenomena at the dome, the overpressure of the internal explosion here is 1.827 times greater than that calculated via Eq. (5). The corners are strongly influenced by the convergence effects of shock waves, so the overpressure of the internal explosion here is 2.72 times greater than that of the calculation via Eq. (5). The overpressure of an internal explosion is affected by reflection phenomena and convergence effects and thus is no longer simply related to the scale distance. Accordingly, the overpressure of shock waves under internal explosions cannot be effectively calculated via empirical formulas.

**Table 4:** Comparison of overpressure at dome and corners of structure.

Location	$\bar {R}/{\text{m}} \cdot {\text{k}}{{\text{g}}^{ - 1/3}}$	ΔP_f_ /MPa	ΔP/MPa	ΔP/ΔP_f_
Dome	3.28	0.0423	0.0773	1.827
Corner	3.05	0.0483	0.1314	2.72

*Notes: $\Delta {P}$ is the overpressure of shock waves under internal explosion, $\Delta {P_f}$ is the overpressure of air blast, and can be determined by Eq. (5).

To visually describe the characteristics of the shock waves of an internal explosion, the reflection amplification factor *A*_pp_ can be defined as the ratio of the overpressure of the internal explosion to the overpressure of the air blast, and the following equation can be obtained.

6 $${A_{{\text{pp}}}}=\Delta P/\Delta {P_f}$$

**Fig. 7:**
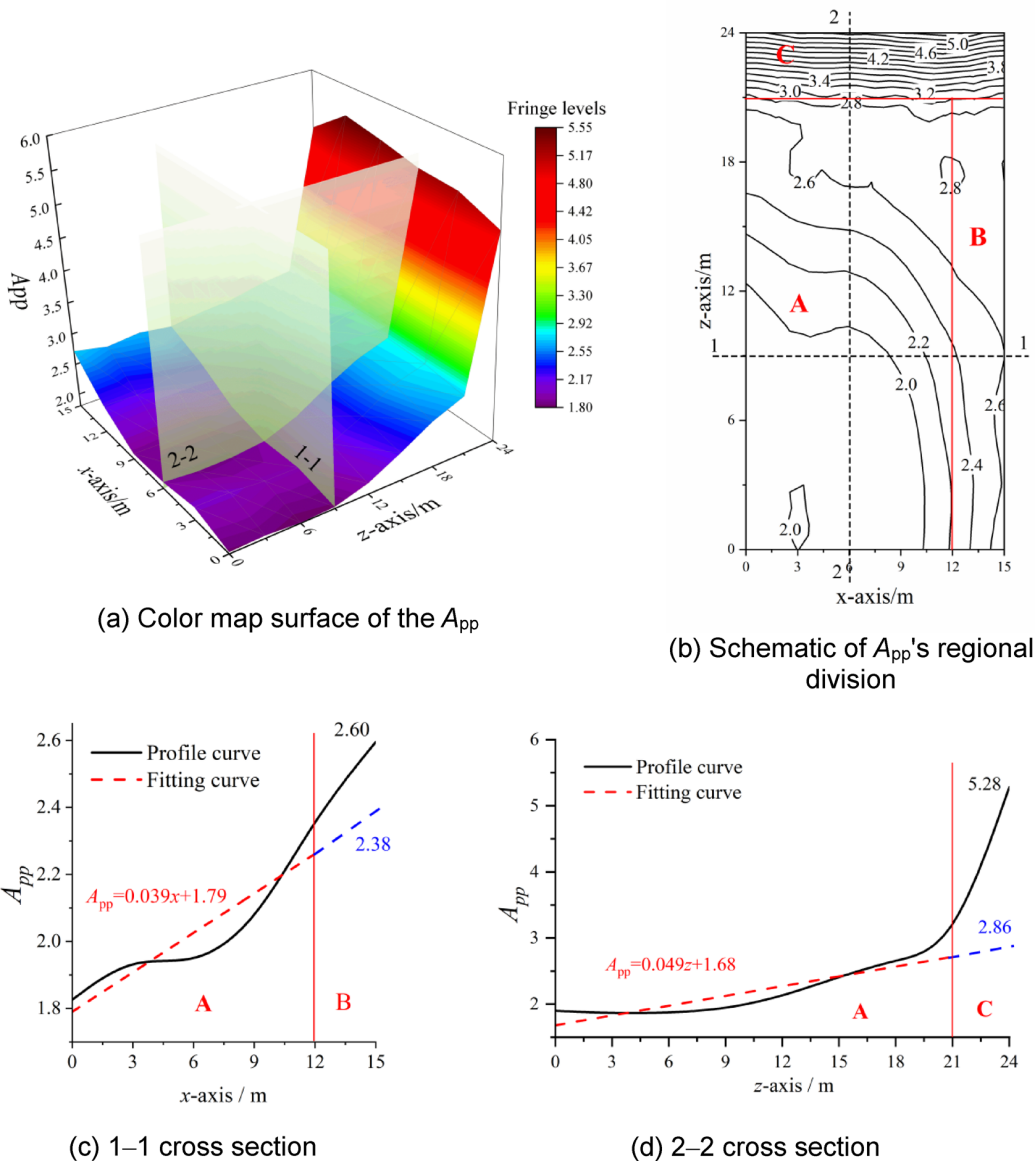
Distribution laws of reflection amplification factor *A*_pp_

The reflection amplification factor *A*_pp_ can be calculated via Eq. (6), and its distribution laws can be plotted. Considering the symmetry of the structure, 1/4 of the distribution sketch is shown in Fig. 7. According to the distribution sketch of the *A*_pp_, the distribution laws of overpressure under internal explosion can be studied.

The 3D surface distribution of the *A*_pp_ is shown in Fig. 7(a), in which the trend of being small in the central region (e.g., *x* = 0 and *z* = 0) and large in the edge regions (e.g., *x* = 15 or *z* = 24) could be visually reflected. The central regions of the *A*_pp_ correspond to the region near the structural dome and are strongly affected by the reflection phenomena of internal explosion. The *A*_pp_ generally increases with increasing *x* and *z*. The edge regions of the *A*_pp_ correspond to the near of the structural corner and are strongly affected by the convergence effects of internal explosion. The *A*_pp_ is notably greater than that for the structural dome and reaches a peak at *z* = 24. The peak *A*_pp_ in the structural corner is approximately three times greater than that in the central region. Thus, the distribution laws of the *A*_pp_ can be divided into zones A, B, and C.

As shown in Fig. 7(b), zones B and C are located 3 m from the edge and are strongly affected by the convergence effects of internal explosions. The remaining region is Zone A, which is strongly affected by the reflection phenomena of the internal explosion.

Figure 7(c) and Fig. 7(d) are the 1–1 cross-section and 2–2 cross-section, respectively. Combining Fig. 7(b), Fig. 7(c) and Fig. 7(d), the *A*_pp_ in Zone A has a regular distribution and can be expressed as a function of *x* and *z*. However, this function cannot effectively predict the *A*_pp_ in Zones B and C. As shown in Fig. 7(c), the *A*_pp_ in Zone B is 2.6, but the predicted result using the function for Zone A is 2.38, which is a decrease of 9.24%. Similarly, as shown in Fig. 7(d), the *A*_pp_ in Zone C is 5.28, but the predicted result using the function for Zone A is 2.86, which is a decrease of 84.61%. The influence of the convergence effects of internal explosion is neglected if the function for Zone A is used to calculate the *A*_pp_ for Zones B and C. Therefore, the *A*_pp_ for Zones A, B, and C should be calculated independently to ensure that the influences of the reflection phenomena and convergence effects on the *A*_pp_ can be fully considered.

### Parametric studies

**Fig. 8:**
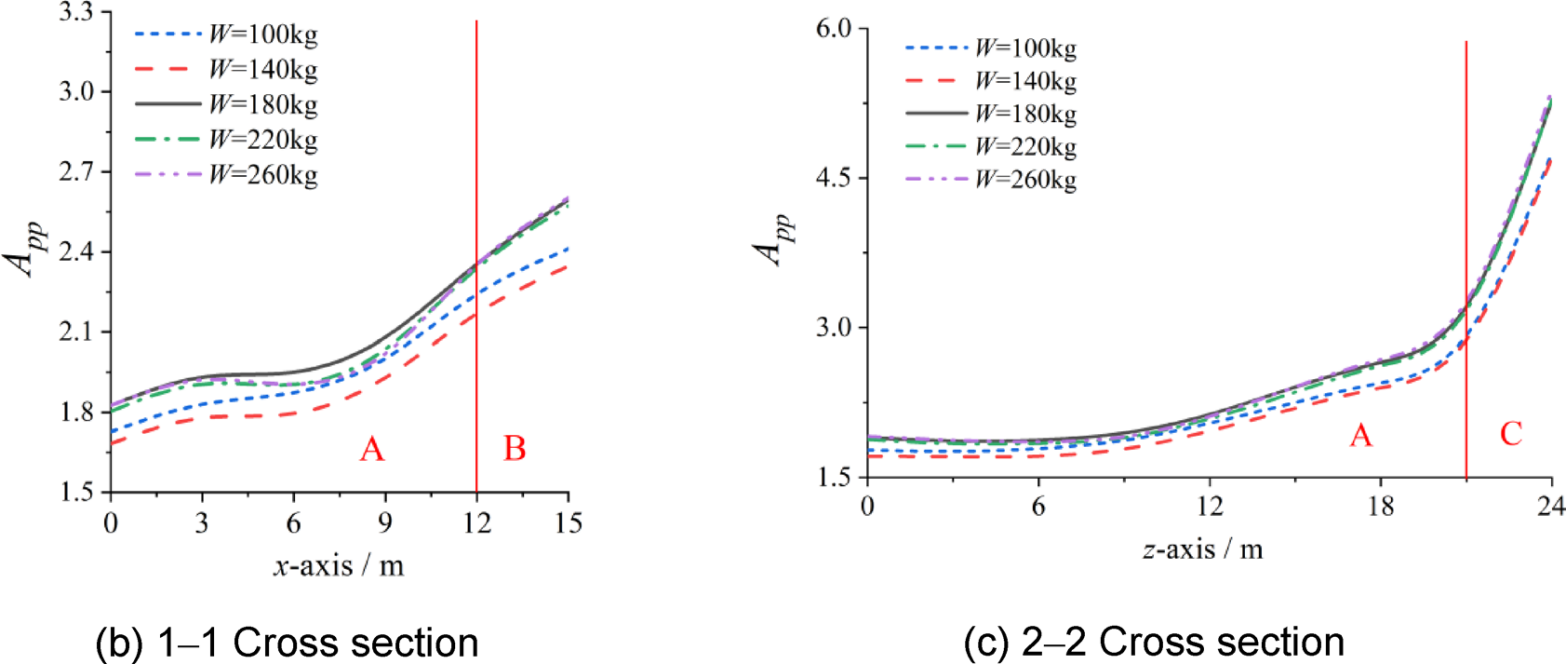
Influence of charge mass *W* on *A*_pp_

**Fig. 9:**
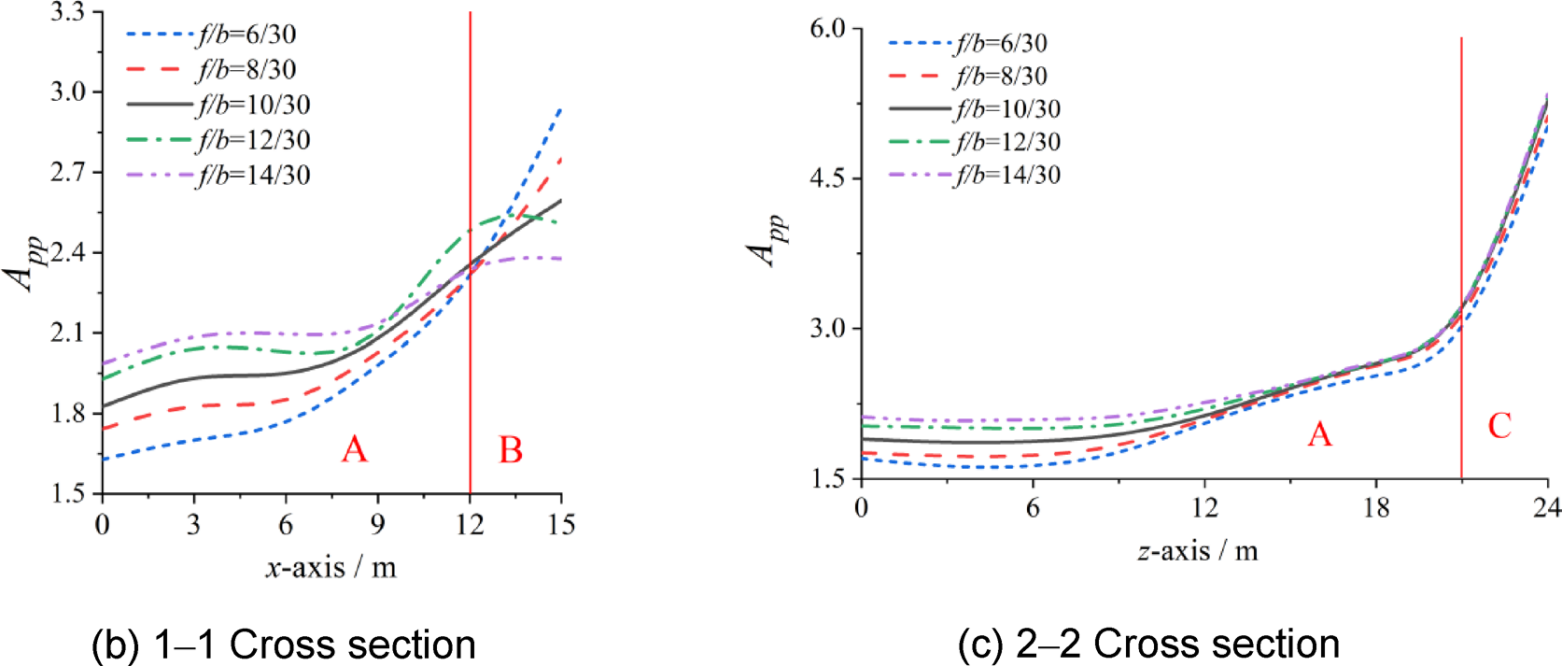
Influence of ratio of rise to span *f/b* on *A*_pp_

**Fig. 10:**
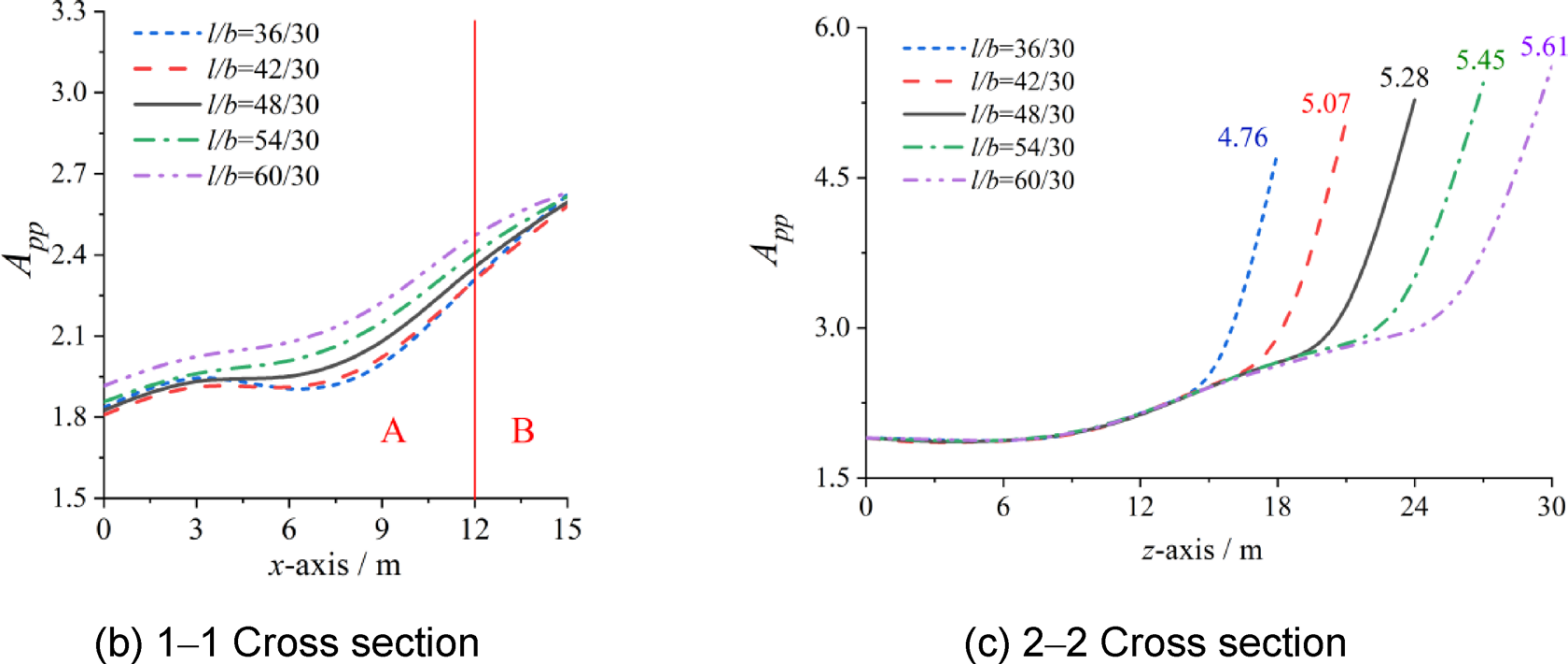
Influence of ratio of length to span *l/b* on *A*_pp_

To study the influences of the charge mass and geometric parameters of the structure on the distribution laws of the *A*_pp_ for Zones A, B, and C, a cylindrical lattice shell with a span of 30 m was taken as an example. The relevant parameters are presented in Table 5, and the *A*_pp_ for Zones A, B, and C were extracted from similar cross-sections in Fig. 7(a) or Fig. 7(b). The results are shown in Figs. 8, 9 and 10.

**Table 5:** Parameters that affect overpressure of shock wave under internal explosion.

Parameters	Value
Charge mass(*W*)	100 kg, 140 kg, 180 kg, 220 kg, 260 kg
Ratio of rise to span(*f/b*)	4/30, 6/30, 8/30, 10/30, 12/30
Ratio of length to span(*l/b*)	36/30, 40/30, 44/30, 48/30, 52/30

As shown in Fig. 8, the influence of the charge mass on the *A*_pp_ can be reflected. Along the *x*-axis and *z*-axis, the trends under different charge masses are the same. The boundary between zones A and B, as well as that between zones A and C, does not change with the charge mass, which all remain 3 m away from the structural edge.

However, the trend of *A*_pp_ fluctuates with changing charge mass, although the fluctuations are not significant. The *A*_pp_ reaches its maximum when the charge mass is 180 kg and reaches its minimum when the charge mass is 140 kg. In Zone A, the differences between the maximum and minimum values are approximately 6–8% along the *x*-axis and approximately 7–10% along the *z*-axis. In zones B and C, the differences between the maximum and minimum values are approximately 9% and 11%, respectively. Therefore, the work condition of the maximum *A*_pp_ can be used in applications, and the influence of the charge mass on the distribution laws of the *A*_pp_ can be ignored. The prediction overpressure of the *A*_pp_ is greater than the practical overpressure of the internal explosion, and there is a certain safety margin within the allowable error range.

As shown in Fig. 9, the influence of the ratio of rise to span on *A*_pp_ can be reflected. Although the boundaries of each zone are still not changed by the ratio of rise to span, the influence of the ratio of rise to span on *A*_pp_ is different in different zones.

In Zone C, the influence of the ratio of the rise to span on *A*_pp_ can be almost neglected. In Zone A, *A*_pp_ increases with increasing ratio of rise to span, but the growth rate gradually slows. For example, at x = 0 m and z = 0 m (i.e., at the dome of the cylindrical lattice shell structure), the ratio of rise to span has the greatest effect on *A*_pp_, and the difference between the maximum and minimum is 18.53%. Near the boundary between zones A and B, as well as between zones A and C, *A*_pp_ is almost invariable.

Unlike those in zones A and C, the *A*_pp_ in Zone B conversely decreases with increasing ratio of rise to span. Zone B is located at the corner where the wall intersects the upper lattice shell. The angle of the intersection can be changed by different ratios of rise to span, and the convergence effects of shock waves under internal explosion may be affected. A larger ratio of rise to span will lead to a larger angle, and then *A*_pp_ will decrease with decreasing convergence effects.

Unlike the charge mass and the ratio of rise to span, the different work conditions of the ratio of length to span could not be compared within the same *z*-axis. However, as shown in Fig. 10, the boundary between zones A and B, as well as that between zones A and C, also remains 3 m away from the structural edge.

As shown in Fig. 10, *A*_pp_ gradually increases with increasing ratio of length to span, but there are differences in Zones A, B, and C. In Zone A, the *A*_pp_ values with different ratios of length to span almost overlap along the *z*-axis. The influence of the ratio of length to span on *A*_pp_ can be neglected along the *z*-axis. However, the influence of the ratio of length to span on *A*_pp_ should be taken seriously along the *x*-axis. The *A*_pp_ in the second half of the *x*-axis is affected mainly by the ratio of length to span.

In contrast to the ratio of rise to span, the *A*_pp_ in Zone B cannot be significantly affected by the ratio of length to span, but the influence of the ratio of length to span on the *A*_pp_ in Zone C should be taken seriously. As shown in Fig. 10(b), the *A*_pp_ in Zone C increases linearly with increasing length-to-span ratio, and the difference between the maximum and minimum values is 15.15%.

## Equivalent model of internal blast loads

To efficiently calculate internal blast loads, it is necessary to establish an equivalent model of internal blast loads on the basis of the results in Sect. "Internal blast loads in cylindrical lattice shell structure". According to Eq. (6), the internal blast loads can be calculated by multiplying the overpressure of the air blast Δ*P*_*f*_ by the reflection amplification factor *A*_pp_, that is:

7 $$\Delta P={A_{{\text{pp}}}} \cdot \Delta {P_f}$$

where Δ*P*_*f*_ can be calculated via Eq. (5).

Therefore, the key to establishing an equivalent model of internal blast loads is to develop a calculation model for the reflection amplification factor *A*_pp_. According to Figs. 8, 9 and 10, the *A*_pp_ has a similar distribution trend, which can be described by a standard distribution and correction factors.

### Standard distribution of model

The distribution of the *A*_pp_ shown in Fig. 7 was used as the standard distribution of the model. As shown in Fig. 7, the standard distribution of the *A*_pp_ was divided into zones A, B and C. According to the analysis results in Sect. "Over-pressure distribution in structure", three zones could be expressed by the linear functions given in Eq. (8).

8 $${A_{{\text{pp}}}}=\gamma +\alpha \cdot x+\beta \cdot z$$

where *γ*, *α*, and *β* are coefficients. The fitting results of the values for each zone are shown in Table 6.

In Zone A, with increasing *x* and *z*, *A*_pp_ shows a gradually increasing trend despite slight fluctuations. To ensure that the equivalent model of internal blast loads has a certain safety margin, the fitting result is taken as the upper limit of the *A*_pp_ in Zone *A.*

Zones B and C are located at the corners where the wall and the lattice shell intersect and are strongly affected by the convergence effects of shock waves under internal explosion. The values of *A*_pp_ in these zones are greater than those in Zone A but with little fluctuation. In other words, the *A*_pp_ values in Zones B and C are not highly correlated with *x* and *z*. To ensure that the equivalent model of internal blast loads has a certain safety margin, the maximum values of the *A*_pp_ in Zones B and C are taken as the representative values for the standard distribution.

**Table 6:** Standard distribution of *A*_pp_

Zones	γ	α	β
A	1.99826	0.01914	0.03459
B	2.87226	0	0
C	5.54170	0	0

### Correction factors of model

According to the parametric studies in Sect. “Parametric studies”, the distribution laws of *A*_pp_ under different conditions were similar. The differences between each work condition can be determined by the correction factor *ξ*. As shown in Eq. (9), *ξ*_*γ*_, *ξ*_*α*_, and *ξ*_*β*_ are the correction factors for the coefficients *γ*, *α*, and *β* in Eq. (8), respectively.

9 $${A_{{\text{pp}}}}={\xi _\gamma } \cdot \gamma +{\xi _\alpha } \cdot \alpha x+{\xi _\beta } \cdot \beta z$$

*ξ*_*γ*_, *ξ*_*α*_, and *ξ*_*β*_ must fully incorporate the combined influences of the charge mass, rise-to-span ratio, and length-to-span ratio. Therefore, three corrector factors can be composed of *ξ*_*W*_, *ξ*_*f/b*_, and *ξ*_*l/b*_, as follows.

10 $$\xi ={\xi _W} \cdot {\xi _{f/b}} \cdot {\xi _{l/b}}$$

where, ${\xi _W}=s \cdot W+t$, ${\xi _{f/b}}=s \cdot (f/b)+t$ and ${\xi _{l/b}}=s \cdot (l/b)+t$. *s* and *t* are the slope and intercept of each correction factor, respectively, and the specific values are shown in Table 7.

**Table 7:** Results of correction factors.

Parameters		A			B	C
		*γ*	*α*	*β*	*γ*	*γ*
${\xi _W}$	s	0	0	0	0	0
	t	1	1	1	1	1
${\xi _{f/b}}$	s	0.8218	6.60893	-2.42931	-0.82857	0
	t	0.74113	-1.25763	1.76464	1.29705	1
${\xi _{l/b}}$	s	0.19514	-1.70585	0	0	0.18962
	t	0.69071	3.70324	1	1	0.6913

## Verification of equivalent model

As seen from Sect. "Equivalent model of internal blast loads", the core of the equivalent model of internal blast loads lies in calculating the reflection amplification factor *A*_pp_ through the standard distribution and the correction factors. In Sect. "Verification of equivalent model", the calculation process is described in detail in two examples, and the rationality and feasibility of the equivalent model of internal blast loads are verified by comparing the model calculation results with the simulation results.

### Case study 1

#### Specifications

A single-layer cylindrical lattice shell structure has a span *b* of 30 m, a length *l* of 39 m, a rise *f* of 7 m, a rise-to-span ratio of 7/30, a length-to-span ratio of 39/30, and a lower support structure height *h* of 10 m. The TNT mass is 200 kg, and the explosive is located at the centre of the structure, 1.5 m above the ground. The overpressure of the shock waves of the structure under internal explosion is calculated via the equivalent model of internal blast loads.

#### Solution

The calculation procedure can be divided into the following four steps.

Step 1. The positions at *x* = 6 m and *z* = 7.3125 m are taken as examples, and the scaled distance $\bar {R}$ and overpressure of the air blast Δ *P*_*f*_ are calculated as follows.

11 $$\begin{gathered} R=\sqrt {{x^2}+{{(y - 1.5)}^2}+{z^2}} =\sqrt {{6^2}+{{14.5576}^2}+{{7.3125}^2}} =17.3608{\text{m}} \hfill \\ \bar {R}=R/\sqrt[3]{W}=17.3608/\sqrt[3]{{200}}{\text{=}}2.9687{\text{m}} \cdot {\text{k}}{{\text{g}}^{ - 1/3}} \hfill \\ \Delta {P_f}=\frac{{0.0317}}{{\bar {R}}}+\frac{{0.3041}}{{{{\bar {R}}^2}}}+\frac{{0.1517}}{{{{\bar {R}}^3}}}{\text{=}}0.0510{\text{MPa}} \hfill \\ \end{gathered}$$

Step 2. The correction factor *ξ* is calculated from the data in Table 7 and Eq. (10), as shown in Table 8.

**Table 8:** Calculation process of correction factors *ξ*.

Parameters and calculation process	A			B	C
	γ	α	β	γ	γ
${\left. {{\xi _W}=s \cdot W+t} \right|_{W=200{\text{kg}}}}$	1	1	1	1	1
${\left. {{\xi _{f/b}}=s \cdot (f/b)+t} \right|_{f/b=7/30}}$	0.9329	0.2845	1.1978	1.1037	1
${\left. {{\xi _{l/b}}=s \cdot (l/b)+t} \right|_{l/b=39/30}}$	0.9444	1.4856	1	1	0.9378
$\xi ={\xi _W} \cdot {\xi _{f/b}} \cdot {\xi _{l/b}}$	0.8810	0.4226	1.1978	1.1037	0.9378

Step 3. The distribution functions of the *A*_pp_ are determined by the standard distribution and correction factors as follows.

12 $$\begin{gathered} {A_{{\text{pp}}}}{\text{=}}{\xi _\gamma }\gamma +{\xi _\alpha }\alpha \cdot x+{\xi _\beta }\beta \cdot z \hfill \\ \begin{array}{*{20}{c}} {}&\begin{gathered} =(0.8810 \times 1.99826)+(0.4226 \times 0.01914) \cdot x+(1.1978 \times 0.03459) \cdot z \hfill \\ {\text{=}}1.7605+0.0081 \cdot x+0.0414 \cdot z \hfill \\ \end{gathered} \end{array} \hfill \\ \end{gathered}$$

13 $${A_{{\text{pp}}}}{\text{=}}{\xi _\gamma }\gamma {\text{=}}1.1037 \times 2.87226{\text{=}}3.1702$$

14 $${A_{{\text{pp}}}}{\text{=}}{\xi _\gamma }\gamma {\text{=}}0.9378 \times 5.54170{\text{=5}}{\text{.1970}}$$

The positions of *x* = 6 m and *z* = 7.3125 m in the cylindrical lattice shell structure are located in Zone A, and *A*_pp_ can be obtained via Eq. (12), as shown below.

15 $$\begin{gathered} {A_{{\text{pp}}}}{\text{=}}1.7605+0.0081 \cdot x+0.0414 \cdot z \hfill \\ \begin{array}{*{20}{c}} {}&{\text{=}} \end{array}1.7605+0.0081 \times 6+0.0414 \times 7.3125 \hfill \\ \begin{array}{*{20}{c}} {}&{{\text{=}}2.112} \end{array} \hfill \\ \end{gathered}$$

Step 4. On the basis of Δ*P*_*f*_ from Step 1 and *A*_pp_ from Step 3, the overpressure of shock waves under internal explosion can be obtained via Eq. (7). At x = 6 m and *z* = 7.3125 m, the overpressure of the shock wave under internal explosion can be calculated as follows:

16 $$\Delta P={A_{{\text{pp}}}} \cdot \Delta {P_f}=2.112 \times 0.051=0.108{\text{MPa}}$$

#### Results

The calculated overpressure of the equivalent model is 0.108 MPa, and the numerical simulation result is 0.094 MPa. The error is 12.63%, which is acceptable for the blast load.

**Fig. 11:**
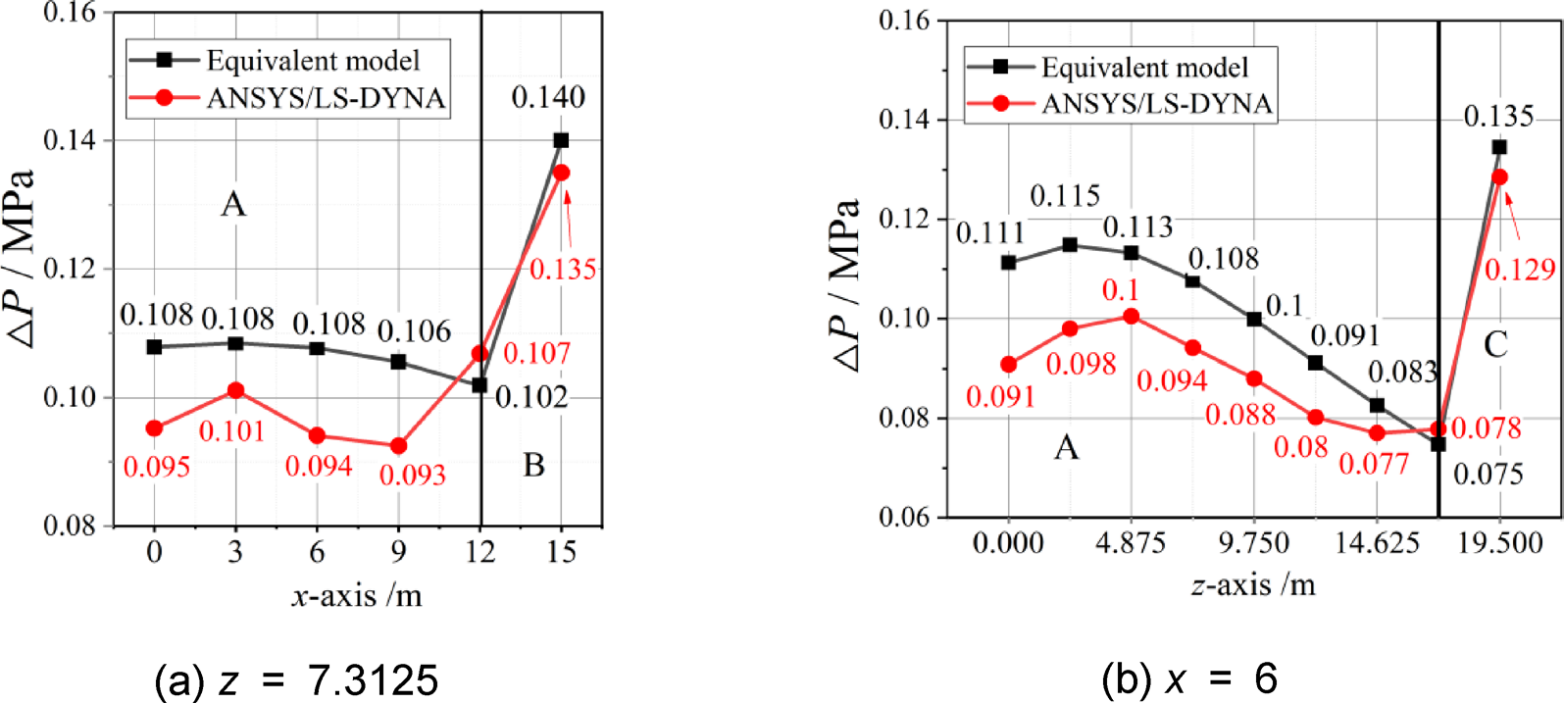
For Example 1, comparison between equivalent model and simulation.

To eliminate any chance of occurrence of individual cases and to verify the reliability of the equivalent model of internal blast loads in Zones B and C, the overpressures of internal explosion along the *x*-axis at *z* = 7.3125 and along the *z*-axis at *x* = 6 were calculated and compared. The results are shown in Fig. 11.

The calculation results of the equivalent model of internal blast loads are generally higher than the numerical simulation results, and the distribution laws are similar to those of the numerical simulation results. In Zone A, the maximum error is 18.38%, occurring at *x* = 6 m and *z* = 0 m. The error gradually decreases with increasing *z*-axis. The minimum error is -4.18%, and the average error is 9.38%. In Zones B and C, the model calculation results are 3.59% and 4.45% greater than the numerical simulation results, respectively. Both a certain safety margin and the characteristics of shock waves under internal explosion can be simultaneously considered by the equivalent model of internal blast loads.

### Case study 2

#### Specifications

A single-layer cylindrical lattice shell structure has a span *b* of 30 m, a length *l* of 51 m, a rise *f* of 12 m, a rise-to-span ratio of 12/30, a length-to-span ratio of 51/30, and a lower support structure height *h* of 10 m. The TNT mass is 160 kg, and the explosive is located at the centre of the structure, 1.5 m above the ground. The overpressure of the shock waves of the structure under internal explosion is calculated via the equivalent model of internal blast loads.

#### Solution

The specific calculation procedure is similar to that in Example 1 and is not repeated here.

#### Results

**Fig. 12:**
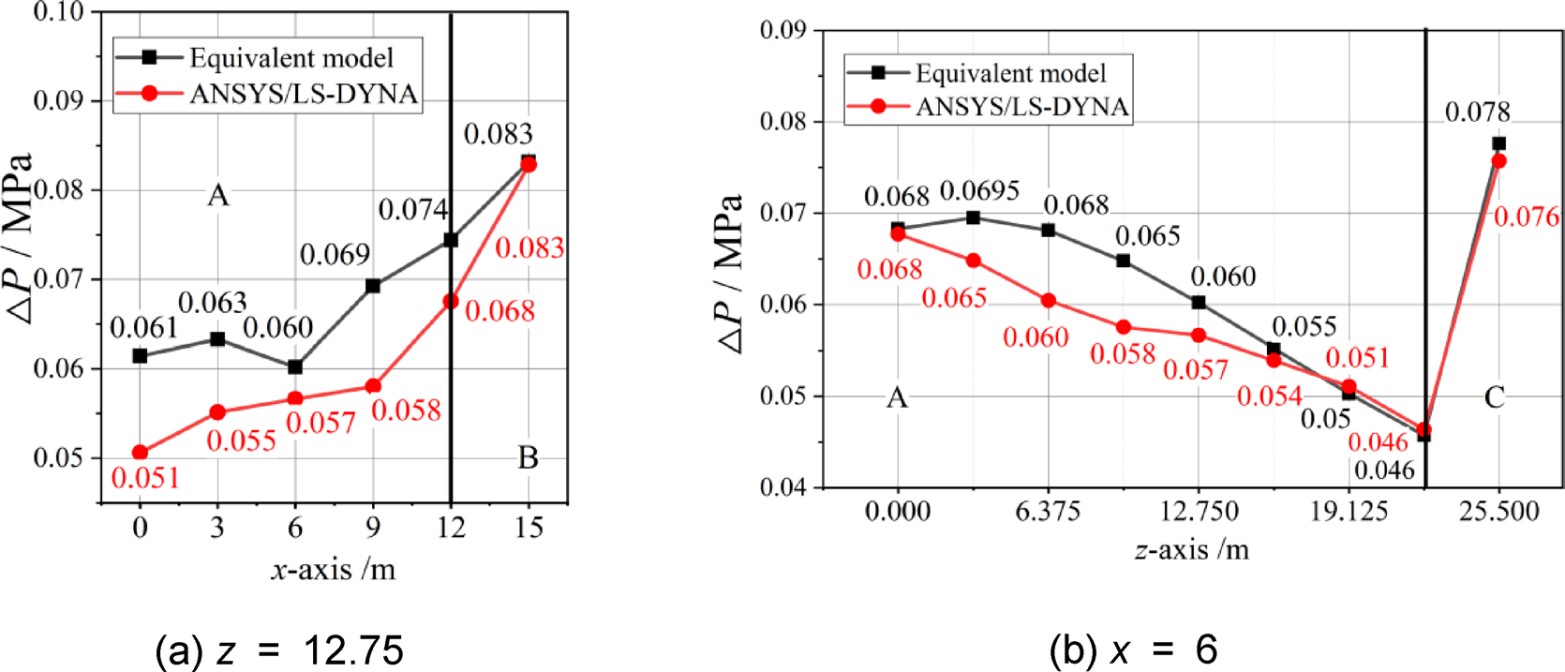
For Example 2, comparison between equivalent model and simulation.

The overpressures of the internal explosion along the *x*-axis at *z* = 12.75 and along the *z*-axis at *x* = 6 were calculated and compared. The results are shown in Fig. 12.

Except for a few individual locations, the calculation results of the equivalent model of internal blast loads are generally higher than the numerical simulation results. In Zone A, the maximum error is 17.66%, the minimum error is -1.41%, and the average error is 7.47%. In Zones B and C, the calculation results of the equivalent model of internal blast loads are 0.083 MPa and 0.078 MPa, respectively, which are greater than the numerical simulation results. The errors are 0.38% and 2.40%, respectively. Combining the calculation and analysis of Example 1, it is demonstrated that there is a stable calculation accuracy for the equivalent model of internal blast loads, which can be applied in practice.

## Conclusions

To improve the calculation efficiency of the internal blast loads of a long-span spatial steel structure in applications, ANSYS/LS-DYNA was used to establish a numerical model of the cylindrical lattice shell structure under internal explosion; propagation laws and overpressure distribution of shock waves under internal explosion were analysed, and the influence of different parameters on the overpressure distribution was discussed. An equivalent model for calculating internal blast loads was established, and the following conclusions were drawn.

(1) A numerical model of an air blast composed of air, ground, explosive and mounting brackets for the sensor was established. The errors between the simulation results and experimental data were less than 8%, so the modelling method and material parameters were shown to be reasonable and reliable.

(2) Shock waves resulting from internal explosions present obvious reflection phenomena and convergence effects, and the overpressure distribution of shock waves resulting from internal explosions is very uneven. Near the structural dome, the overpressure of the internal explosion was strongly affected by the reflection phenomenon, which was marked as Zone A. At the corners of the structure, the overpressure of the internal explosion was strongly affected by the convergence effects, which were, respectively marked as Zones B and C. The boundaries between Zones A and B or Zone C were located approximately 3 m away from the corner of the structure.

(3) The reflection amplification factor *A*_pp_ is the ratio of the overpressure of an internal explosion to that of an air blast, which reflects the characteristics of the overpressure distribution of shock waves under an internal explosion well. The standard distribution in the equivalent model was a function of *A*_pp_ with respect to the structural position coordinates. Therefore, the reflection phenomenon and convergence effects of shock waves under internal explosion were fully considered in the equivalent model.

(4) The application results of two engineering examples revealed that the calculation results of the equivalent model were similar to those of the numerical simulation and were generally greater than those of the numerical simulation. In other words, the equivalent model for calculating internal blast loads not only has calculation accuracy but also has a certain safety redundancy in applications.

(5) Notably, the equivalent model was suitable for calculating the internal blast loads of cylindrical lattice shell structures with a length‒span ratio between 1.2 and 1.73, a rise‒span ratio between 0.13 and 0.4, and no venting explosion.

## Data Availability

The datasets used and/or analyzed during the current study available from the corresponding author on reason able request.
